# Supplementary material for: Optochemical control of slow-wave sleep in the nucleus accumbens of male mice by a photoactivatable allosteric modulator of adenosine A2A receptors
Source: Nat Commun. 2024 Apr 30;15:3661. doi: 10.1038/s41467-024-47964-4 (PMC11061178; doi:10.1038/s41467-024-47964-4)
Supplement: Supplementary file 1 — Supplementary Information [file 41467_2024_47964_MOESM1_ESM.pdf]

Optochemical control of slow-wave sleep in the nucleus accumbens of male mice by a photoactivatable allosteric modulator of adenosine A_{2A} receptors

Supplementary Information

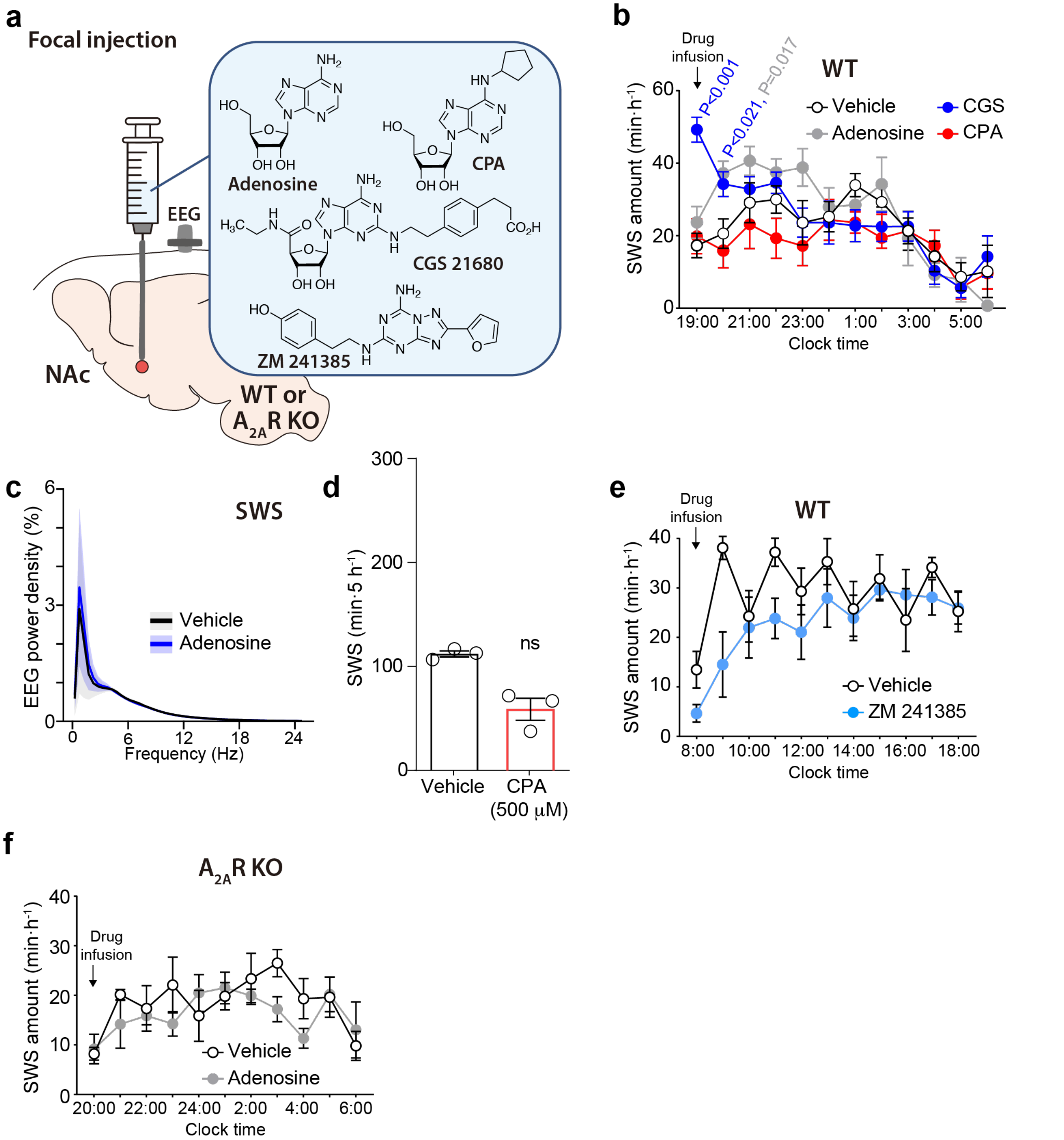

Supplementary Figure 1. **Pharmacologic activation of NAc by focal injection of adenosine and other drugs. a** Schematic of pharmacologic activation of NAc by focal injection of adenosine, CGS 21280, CPA, or ZM 241385 into freely behaving WT and A_{2A}R KO mice, illustrated by Sara Kobayashi. **b** Time courses of SWS after focal NAc injection of adenosine, CGS 21280, or CPA into freely behaving WT. **c** EEG power density of SWS during the injection of vehicle or adenosine. **d** Total amount of SWS for 5 h after focal drug injection of highly concentrated CPA into the NAc. **e**, **f** Time courses of SWS after focal NAc injection with ZM 241385 into freely behaving WT (**e**) and adenosine

into A_{2A}R KO mice (**f**). Data [n=6 (vehicle), 4 (adenosine), 6 (CGS), and 6 (CPA) biologically independent animals] are presented as mean ± SEM. Unpaired 2-tailed t-test compared with the vehicle injections. Source data have been deposited in the Figshare database [https://doi.org/10.6084/m9.figshare.25468084]. Abbreviations: A_{2A}R, adenosine A_{2A} receptor; EEG, electroencephalogram; KO, knockout; NAc, nucleus accumbens; ns, not significant; SEM, standard error of the mean; SWS, slow-wave sleep; WT, wild type.

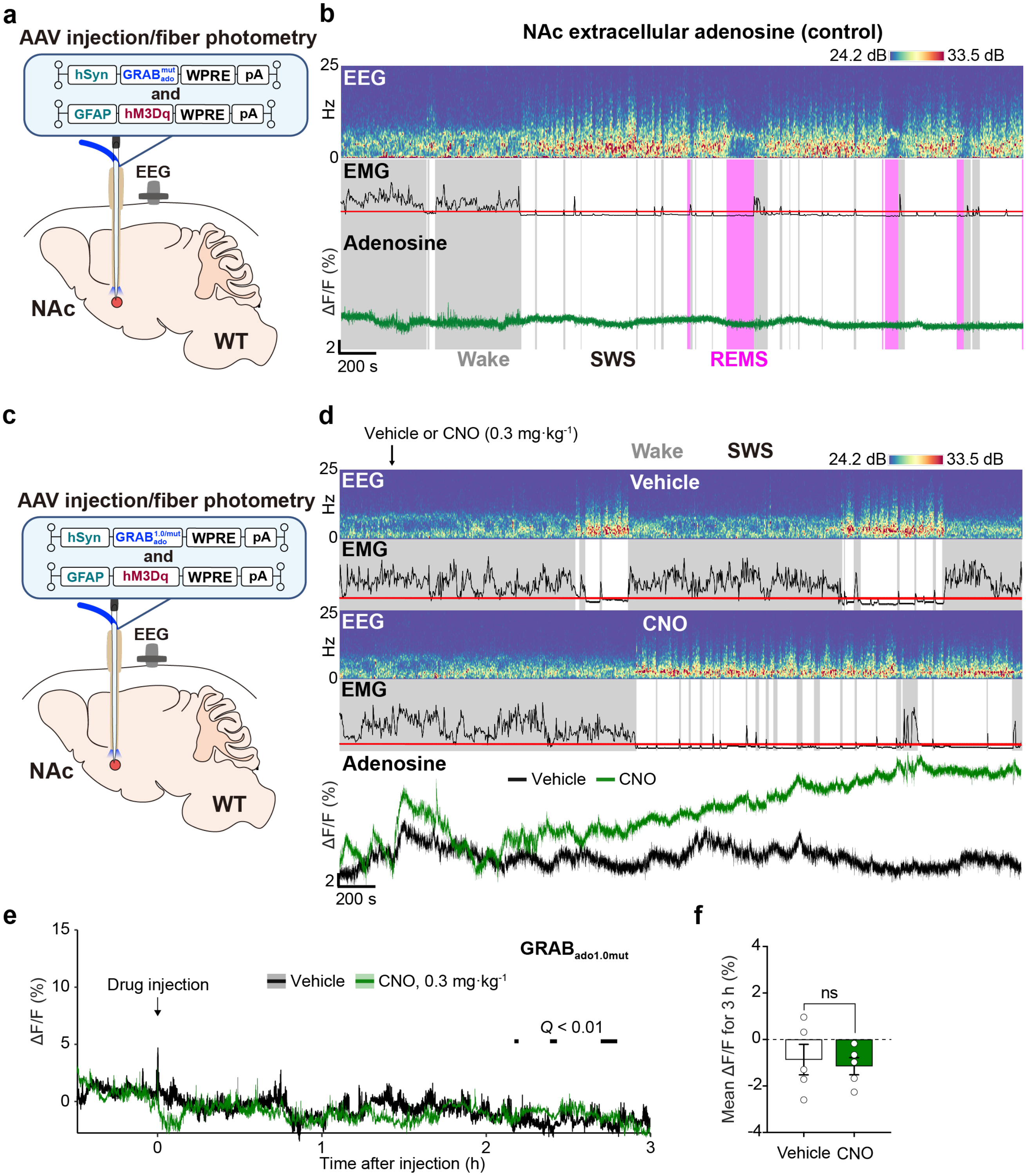

Supplementary Figure 2. **Ado1.0 and Ado1.0mut signals in the NAc. a** Schematic of microinjection of AAVs expressing neuronal Ado1.0mut and astrocytic hM3Dq DREADD and optic fiber placement in the NAc of WT mice, illustrated by Sara Kobayashi. **b** Typical examples of EEG, EMG, and adenosine signals for 1 h in the NAc of a WT mouse after injecting an AAV expressing Ado1.0mut. **c** Schematic of microinjection of AAVs expressing neuronal Ado1.0 or Ado1.0mut and astrocytic

hM3Dq DREADD and optic fiber placement in the NAc of WT mice, illustrated by Sara Kobayashi. **d** Typical examples of EEG, EMG, and adenosine signals for 1 h in the NAc of a WT mouse after chemogenetic stimulation of astrocytes of mice injected with AAVs expressing neuronal Ado1.0 and astrocytic hM3Dq DREADD. The EMG traces are shown as root mean square and the red lines indicate level 5. **e**, **f** Time course (**e**) and mean (**f**) signals of Ado1.0mut after chemogenetic stimulation of astrocytes in the NAc of WT. Data (n=5 biologically independent animals/group) are presented as mean ± SEM [shaded areas in (**e**) or error bars in (**f**)]. **e** Horizontal bars indicate false discovery rate *Q*. Source data have been deposited in the Figshare database [https://doi.org/10.6084/m9.figshare.25468084]. Abbreviations: AAV, adeno-associated virus; CNO, clozapine N-oxide; DREADD, designer receptors exclusively activated by designer drugs; EEG, electroencephalogram; EMG, electromyogram; GFAP, glial fibrillary acidic protein; $GRAB_{Ado1.0/mut}$, G protein-coupled receptor-activation-based adenosine sensor Ado1.0 or non-binding mutant Ado1.0mut; hSyn, human synapsin; NAc, nucleus accumbens; ns, not significant; pA, polyadenylation signal; REMS, rapid eye movement sleep; SEM, standard error of the mean; SWS, slow-wave sleep; WPRE, woodchuck hepatitis virus posttranscriptional regulatory element; WT, wild type.

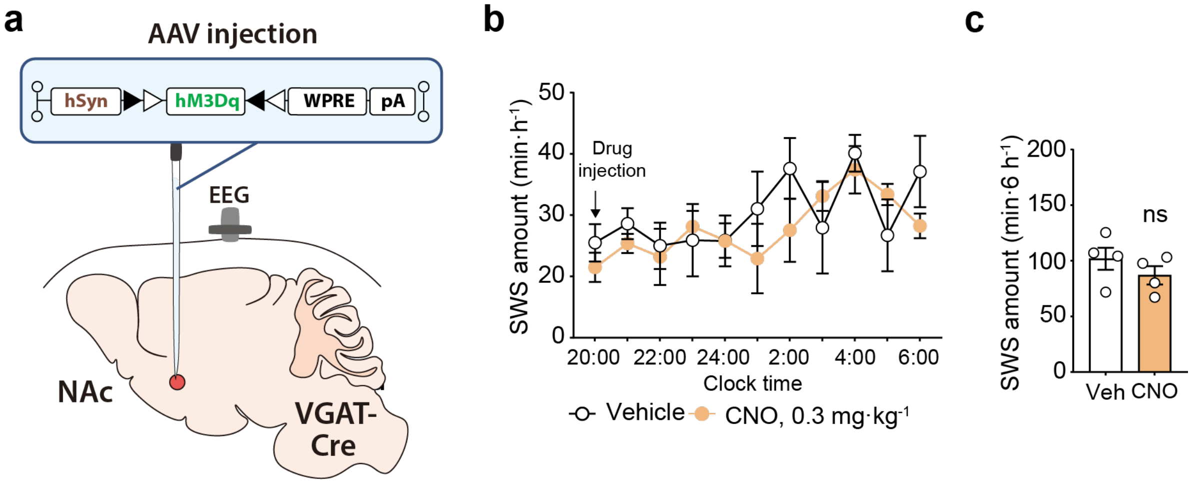

Supplementary Figure 3. **Chemogenetic activation of NAc GABAergic medium spiny neurons. a** Schematic of microinjection of an AAV expressing Cre-dependent hM3Dq DREADD into the NAc of VGAT-Cre mice to direct DREADD expression to GABAergic medium spiny neurons, illustrated by Sara Kobayashi. **b**, **c** Time course (**b**) and total SWS amount for 6 h (**c**) after chemogenetic stimulation of GABAergic neurons in the NAc. Data (n=4 biologically independent animals/group) are presented as mean ± SEM. Source data have been deposited in the Figshare database [https://doi.org/10.6084/m9.figshare.25468084]. Abbreviations: AAV, adeno-associated virus; CNO, clozapine N-oxide; Cre, Cre recombinase; DREADD, designer receptors exclusively activated by designer drugs; EEG, electroencephalogram; hSyn, human synapsin; NAc, nucleus accumbens; ns, not significant; pA, polyadenylation signal; SEM, standard error of the mean; SWS, slow-wave sleep; VGAT, vesicular γ-aminobutyric acid transporter; WPRE, woodchuck hepatitis virus posttranscriptional regulatory element.

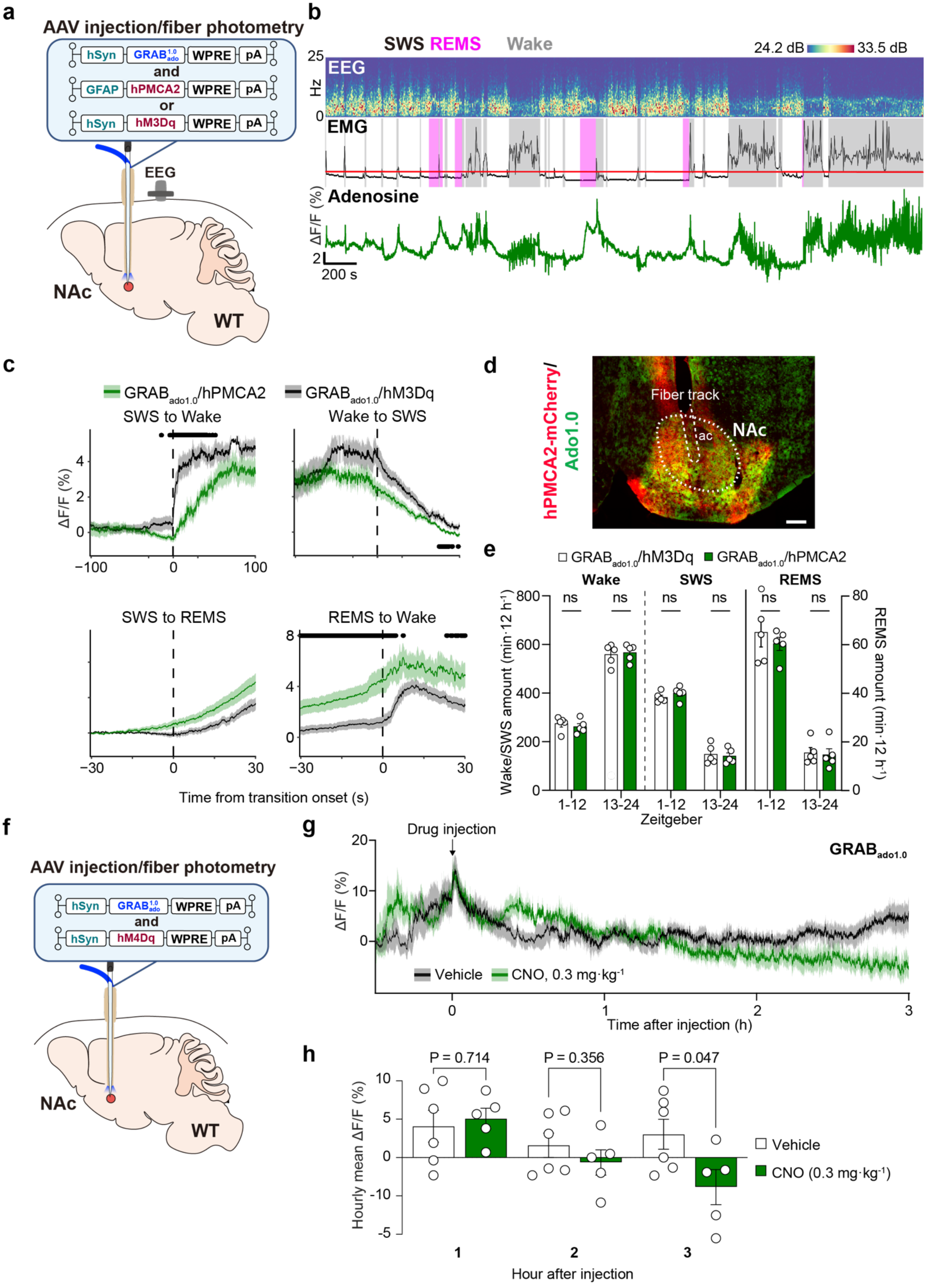

a
AAV injection/fiber photometry
hSyn GRAB ado 1.0 WPRE pA
and
GFAP hPMCA2 WPRE pA
or
hSyn hM3Dq WPRE pA
EEG
NAc
WT
b
SWS REMS Wake
24.2 dB 33.5 dB
EEG
EMG
Adenosine
ΔF/F (%)
200 s
c
GRAB ado1.0/hPMCA2
GRAB ado1.0/hM3Dq
SWS to Wake
Wake to SWS
SWS to REMS
REMS to Wake
Time from transition onset (s)
d
hPMCA2-mCherry/
Ado1.0
Fiber track
ac
NAc
e
GRAB ado1.0/hM3Dq
GRAB ado1.0/hPMCA2
Wake
SWS
REMS
ns
Wake/SWS amount (min·12 h⁻¹)
REMS amount (min·12 h⁻¹)
1-12
13-24
Zeitgeber
f
AAV injection/fiber photometry
hSyn GRAB ado 1.0 WPRE pA
and
hSyn hM4Dq WPRE pA
NAc
WT
g
Drug injection
GRAB ado1.0
ΔF/F (%)
Vehicle
CNO, 0.3 mg·kg⁻¹
Time after injection (h)
h
P = 0.714
P = 0.356
P = 0.047
Hourly mean ΔF/F (%)
Vehicle
CNO (0.3 mg·kg⁻¹)
Hour after injection

Supplementary Figure 4. **Inhibition of NAc astrocytes or neurons partially affects extracellular adenosine levels. a** Schematic of microinjection of AAVs expressing neuronal Ado1.0 and astrocytic hPMCA2 or Ado1.0 and hM3Dq DREADD and optic fiber placement in the NAc of WT mice, illustrated by Sara Kobayashi. **b** Typical examples of EEG, EMG, and adenosine signals for 1 h in the NAc of a WT mouse after injecting AAVs expressing Ado1.0 and hPMCA2. The EMG traces are shown as root mean square and the red lines indicate level 5 activity. **c** Mean NAc adenosine signals before and after each state transition. Data for Ado1.0/hPMCA2 [Transitions examined over 5 independent experiments: n=29 (Wake-SWS), n=27 (SWS-Wake), n=35 (SWS-REMS), and n=16 (REMS-SWS)] are presented as mean ± SEM (shaded areas) and compared to Ado1.0/hM3Dq data in Fig. 2c. •P<0.01 between hPMCA2 and hM3Dq DREADD-expressing mice. **d** Immunostaining for mCherry together with Ado1.0 fluorescence in WT mice after injecting AAVs expressing Ado1.0 and hPMCA2. Scale bar: 500 µm. **e** Total amount of Wake, SWS, and REMS during the light (ZT 1-12) and dark (ZT 13-24) periods after hPMCA-mediated hyperpolarization of NAc astrocytes in WT mice. Data (n=5 biologically independent animals/group) are presented as mean ± SEM. **f** Schematic of microinjection of AAVs expressing neuronal Ado1.0 and hM4Di DREADD and optic fiber placement in the NAc of WT mice, illustrated by Sara Kobayashi. **g**, **h** Time course (**g**) and mean (**h**) adenosine levels after chemogenetic inhibition of neurons in the NAc of WT. Data [n=6 (vehicle) and n=5 (CNO) biologically independent animals in each group] are presented as mean ± SEM [shaded areas in (**g**) or error bars in (**h**)]. Source data have been deposited in the Figshare database [https://doi.org/10.6084/m9.figshare.25468084]. Abbreviations: AAV, adeno-associated virus; CNO, clozapine N-oxide; DREADD, designer receptors exclusively activated by designer drugs; EEG, electroencephalogram; EMG, electromyogram; GFAP, glial fibrillary acidic protein; $\text{GRAB}_{\text{Ado1.0/mut}}$, G protein-coupled receptor-activation-based adenosine sensor Ado1.0; hPMCA2, human plasma membrane calcium pump isoform type 2; hSyn, human synapsin; NAc, nucleus accumbens; pA, polyadenylation signal; REMS, rapid eye movement sleep; SEM, standard error of the mean; SWS, slow-wave sleep; WPRE, woodchuck hepatitis virus posttranscriptional regulatory element; WT, wild type; ZT, Zeitgeber.

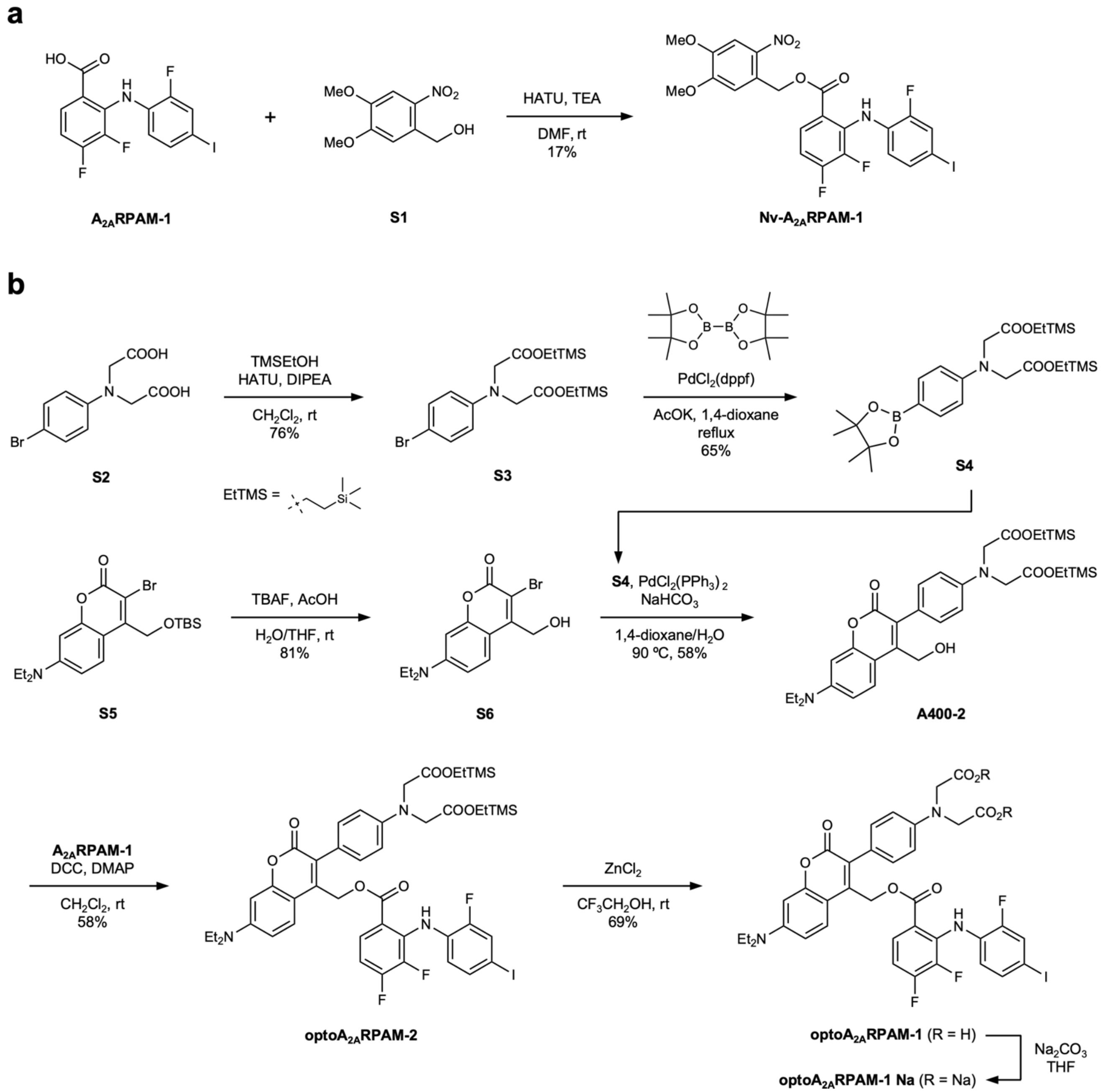

Supplementary Figure 5. **Chemical synthesis of photoactivatable positive allosteric modulators of A_{2A}Rs. a** Nv-A_{2A}RPAM-1 was synthesized by condensation of the carboxyl group of A_{2A}RPAM-1 with the hydroxyl group of 2-nitroveratrole alcohol. **b** OptoA_{2A}RPAM-1 and optoA_{2A}RPAM-2 were synthesized by condensation of the carboxyl group of A_{2A}RPAM-1 with the hydroxyl group of the coumarin derivative A400 in several steps. Abbreviations: AcOH, acetic acid; AcOK, potassium acetate; DCC, *N*,*N*'-dicyclohexylcarbodiimide; DIPEA, *N*,*N*-diisopropylethylamine; DMAP, 4-dinethylaminopyridine; DMF, *N,N*-dimethylformamide; EtTMS, Trimethylsilylethanol derivative; HATU, 1-[bis(dimethylamino)methylene]-1H-1,2,3-triazolo[4,5-b]pyridinium 3-oxide hexafluorophosphate; $PdCl_2(PPh_3)_2$, bis(triphenylphosphine)palladium(II) dichloride; $PdCl_2$(dppf),

[1,1′-bis(diphenylphosphino)ferrocene]dichloropalladium(II); rt, room temperature; TBAF, tetra-*n*-butylammonium fluoride; TEA, triethylamine; THF, tetrahydrofuran; TMSEtOH, trimethylsilylethanol.

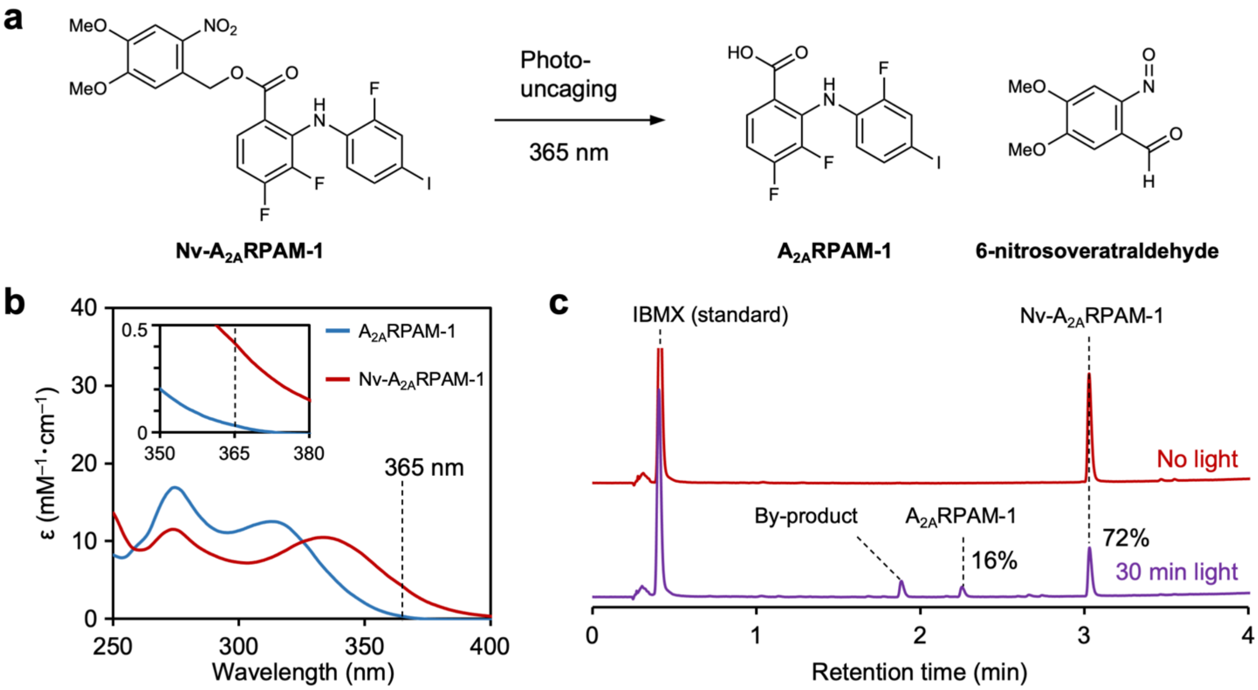

Supplementary Figure 6. **Generation of photoactivatable Nv-A$_{2A}$R PAM. a** Schematic of photo-uncaging of Nv-A$_{2A}$RPAM-1 by UV light (365 nm). **b** UV–visible spectrum of A$_{2A}$RPAM-1 and Nv-A$_{2A}$RPAM-1. (c) UPLC analysis of Nv-A$_{2A}$RPAM-1 before and after UV light irradiation. Abbreviation: IBMX, isobutylmethylxanthine; UPLC, ultra-high-performance liquid chromatography; UV, ultraviolet.

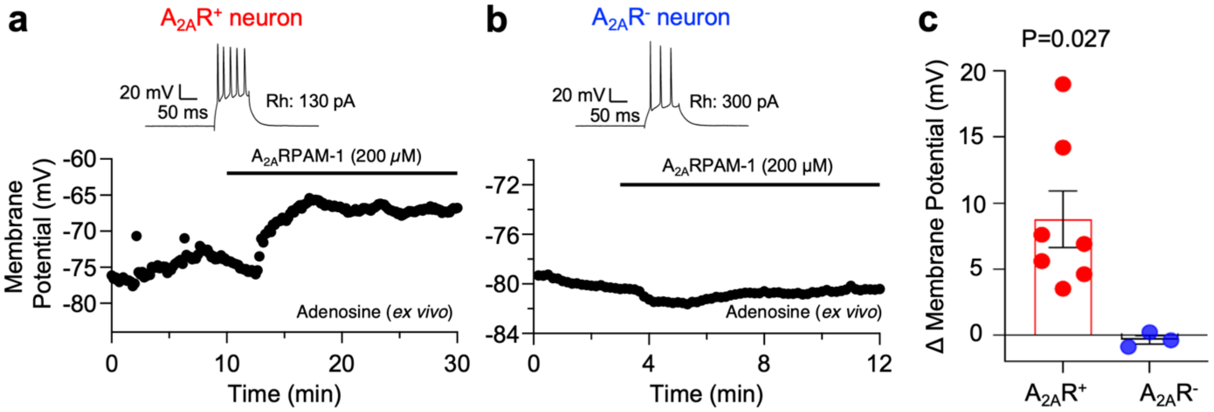

Supplementary Figure 7. **Whole-cell patch-clamp electrophysiology of NAc neurons treated with A_{2A}RPAM-1.** **a**, **b** Resting membrane potential of NAc $A_{2A}R^+$ (**a**) and $A_{2A}R^-$ (**b**) neurons after treatment with A_{2A}RPAM-1. The rheobase (Rh) was used to identify $A_{2A}R^+$ (low Rh) or $A_{2A}R^-$ (high Rh) neurons (upper traces in **a** and **b**). **c** Changes in the membrane potential of NAc $A_{2A}R^+$ and $A_{2A}R^-$ neurons in WT mice by allosteric activation with A_{2A}RPAM-1. Data [n=7 ($A_{2A}R^+$) and n=3 ($A_{2A}R^-$) biologically independent animals in each group] are presented as mean ± SEM. Source data have been deposited in the Figshare database [https://doi.org/10.6084/m9.figshare.25468084]. Abbreviations: A_{2A}R, adenosine A_{2A} receptor; NAc, nucleus accumbens; SEM, standard error of the mean; WT, wild-type.

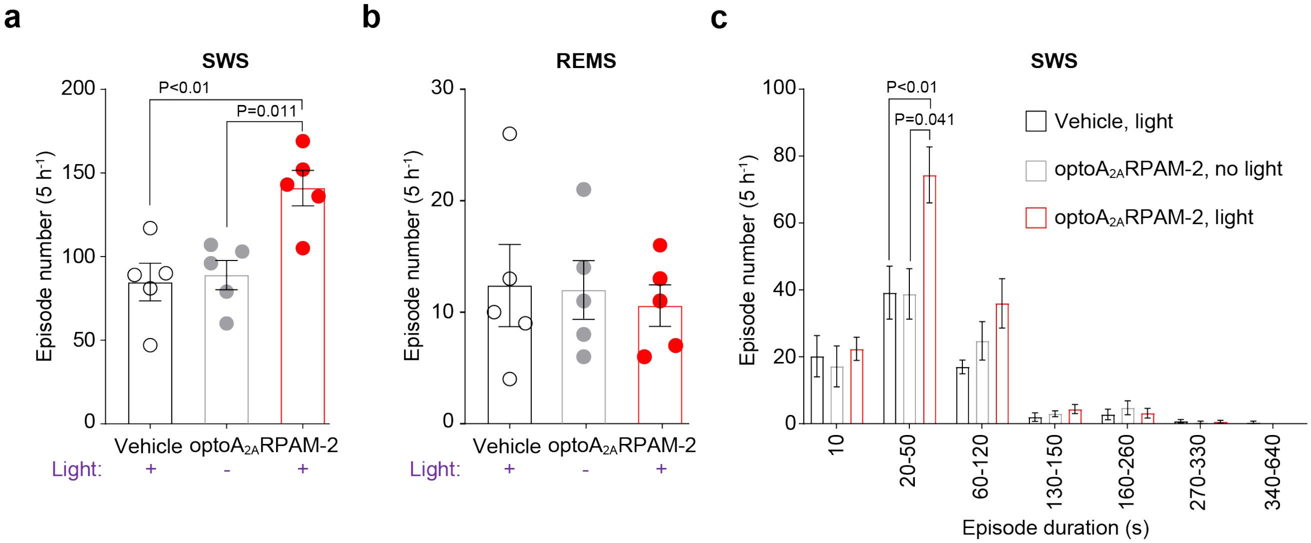

Supplementary Figure 8. **Changes in sleep architecture after systemic administration of optoA$_{2A}$RPAM-2 together with NAc photoirradiation. a-c** Mean episode number of SWS (**a**) and REMS (**b**) and SWS episode spectrum (**c**) after administration of vehicle or optoA$_{2A}$RPAM-2 together with NAc photoirradiation. Data (n=5 biologically independent animals/group) are presented as mean ± SEM. Source data have been deposited in the Figshare database [https://doi.org/10.6084/m9.figshare.25468084]. Abbreviations: NAc, nucleus accumbens; REMS, rapid eye movement sleep; SEM, standard error of the mean; SWS, slow-wave sleep.

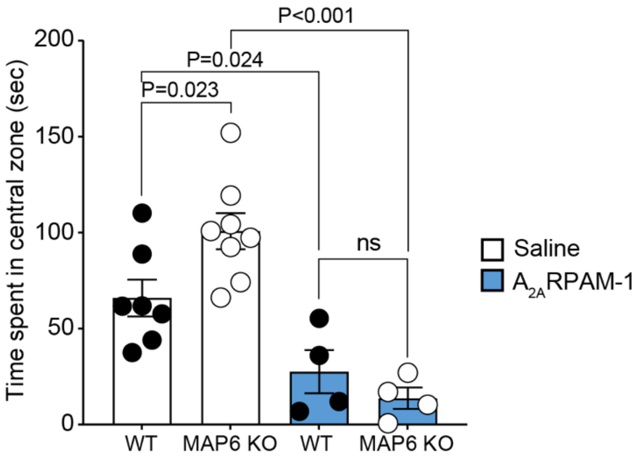

Supplementary Figure 9. **Open field test with WT and microtubule-associated protein 6 (MAP6) KO mice after treatment with A_{2A}RPAM-1.** Time spent in the central zone of the open field. Data [n=7 (WT/saline), n=8 (MAP6 KO/saline), and n=4 (WT/A_{2A}RPAM-1 or MAP6 KO/A_{2A}RPAM-1) biologically independent animals in each group] are presented as mean ± SEM. Source data have been deposited in the Figshare database [https://doi.org/10.6084/m9.figshare.25468084]. Abbreviations: KO, knockout; MAP6, microtubule-associated protein 6; SEM, standard error of the mean; WT, wild type.

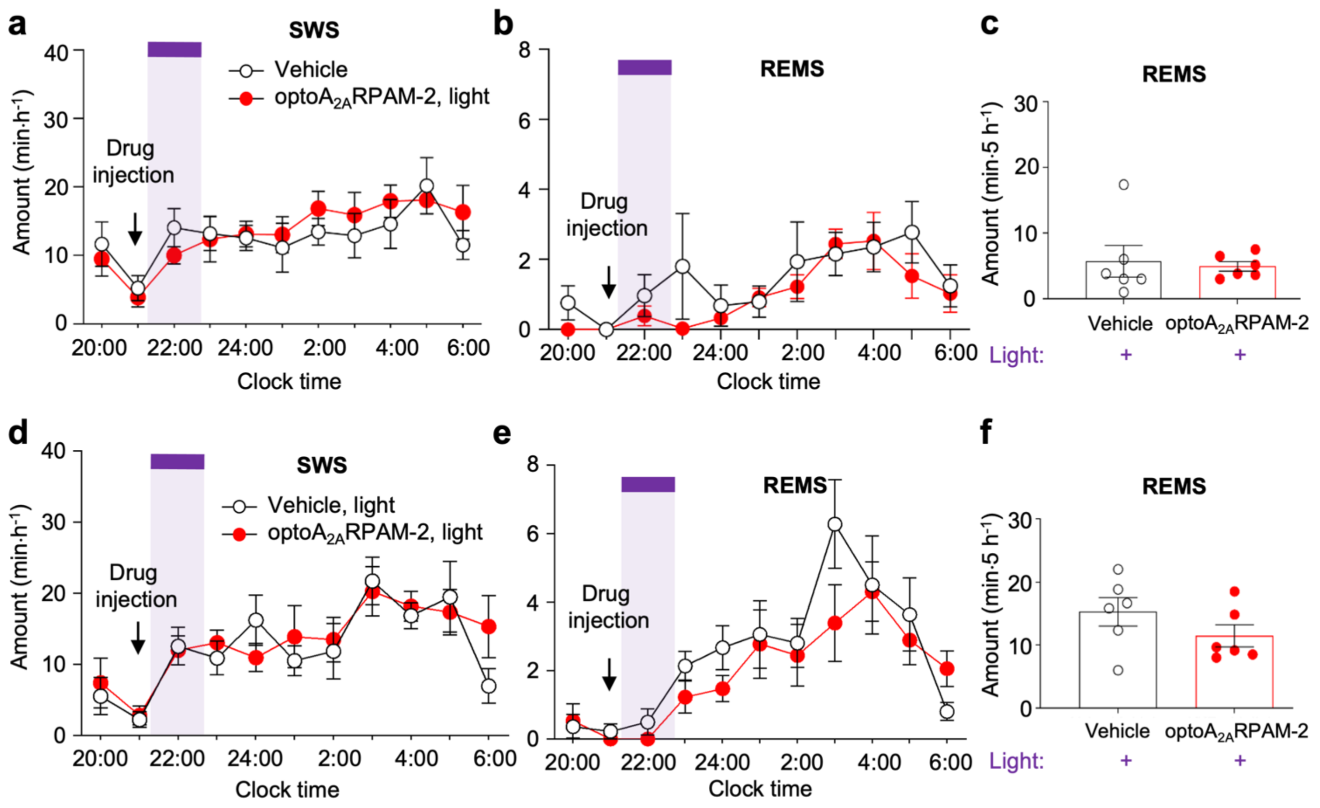

Supplementary Figure 10. **OptoA$_{2A}$RPAM-2 optochemical stimulation of the NAc in A$_{2A}$R KO mice and the VLPO in WT mice. a**, **b** Time course of SWS (**a**) and REMS (**b**) after optoallosteric NAc activation with optoA$_{2A}$RPAM-2 in A$_{2A}$R KO mice. **c** Total amount of REMS for 5 h after optoallosteric NAc activation with optoA$_{2A}$RPAM-2 in A$_{2A}$R KO mice. **d**, **e** Time course of SWS (**d**) and REMS (**e**) after optoallosteric VLPO activation with optoA$_{2A}$RPAM-2 in WT mice. **f** Total amount of REMS for 5 h after optoallosteric VLPO activation with optoA$_{2A}$RPAM-2 in WT mice. Data (n=6 biologically independent animals/group) are presented as mean ± SEM. **a**, **b**, **d**, **e** The purple bar indicates 1-h light illumination. Source data have been deposited in the Figshare database [https://doi.org/10.6084/m9.figshare.25468084]. Abbreviations: A$_{2A}$R, adenosine A$_{2A}$ receptor; EEG, electroencephalogram; EMG, electromyogram; KO, knockout; NAc, nucleus accumbens; REMS, rapid eye movement sleep; SEM, standard error of the mean; SWS, slow-wave sleep; VLPO, ventrolateral preoptic area; WT, wild type.
